# Efficacy of 12 weeks oral beta‐alanine supplementation in patients with chronic obstructive pulmonary disease: a double‐blind, randomized, placebo‐controlled trial

**DOI:** 10.1002/jcsm.13048

**Published:** 2022-08-17

**Authors:** Jana De Brandt, Wim Derave, Frank Vandenabeele, Pascal Pomiès, Laura Blancquaert, Charly Keytsman, Marina S. Barusso‐Grüninger, Fabiano F. de Lima, Maurice Hayot, Martijn A. Spruit, Chris Burtin

**Affiliations:** ^1^ Faculty of Rehabilitation Sciences, REVAL ‐ Rehabilitation Research Center Hasselt University Diepenbeek Belgium; ^2^ BIOMED ‐ Biomedical Research Institute Hasselt University Diepenbeek Belgium; ^3^ Department of Movement and Sports Sciences Ghent University Ghent Belgium; ^4^ PhyMedExp University of Montpellier – INSERM – CNRS – CHRU Montpellier Montpellier France; ^5^ LEFiR ‐ Spirometry and Respiratory Laboratory São Carlos Federal University ‐ UFSCar São Carlos São Paulo Brazil; ^6^ Faculty of Science and Technology, Department of Physical Therapy, Postgraduate Program in Physical Therapy São Paulo State University (UNESP) Presidente Prudente São Paulo Brazil; ^7^ Department of Research and Education CIRO+ Horn The Netherlands; ^8^ Department of Respiratory Medicine, NUTRIM School of Nutrition and Translational Research in Metabolism Maastricht University Medical Centre Maastricht The Netherlands

**Keywords:** Carnosine, Chronic obstructive pulmonary disease, Physical capacity, Oxidative/carbonyl stress

## Abstract

**Background:**

Beta‐alanine (BA) supplementation increases muscle carnosine, an abundant endogenous antioxidant and pH buffer in skeletal muscle. Carnosine loading promotes exercise capacity in healthy older adults. As patients with chronic obstructive pulmonary disease (COPD) suffer from elevated exercise‐induced muscle oxidative/carbonyl stress and acidosis, and from reduced muscle carnosine stores, it was investigated whether BA supplementation augments muscle carnosine and induces beneficial changes in exercise capacity, quadriceps function, and muscle oxidative/carbonyl stress in patients with COPD.

**Methods:**

In this double‐blind, randomized, placebo (PL)‐controlled trial (clinicaltrials.gov identifier: NCT02770417), 40 patients (75% male) with COPD (mean ± standard deviation: age 65 ± 6 years; FEV_1_% predicted 55 ± 14%) were assigned to 12 weeks oral BA or PL supplementation (3.2 g/day). The primary outcome, i.e. muscle carnosine, was quantified from m. vastus lateralis biopsies obtained before and after intervention. Co‐primary outcomes, i.e. incremental and constant work rate cycle capacity, were also assessed. Linear mixed model analyses were performed. Compliance with and side effects of supplement intake and secondary outcomes (quadriceps strength and endurance, and muscle oxidative/carbonyl stress) were also assessed.

**Results:**

Beta‐alanine supplementation increased muscle carnosine in comparison with PL in patients with COPD (mean difference [95% confidence interval]; +2.82 [1.49–4.14] mmol/kg wet weight; *P* < 0.001). Maximal incremental cycling capacity (VO_2_peak: +0.5 [−0.7 to 1.7] mL/kg/min; *P* = 0.384, Wpeak: +5 [−1 to 11] W; *P* = 0.103) and time to exhaustion on the constant work rate cycle test (+28 [−179 to 236] s; *P* = 0.782) did not change significantly. Compliance with supplement intake was similar in BA (median (quartile 1–quartile 3); 100 (98–100)%) and PL (98 (96–100)%) (*P* = 0.294) groups, and patients did not report side effects possibly related to supplement intake. No change was observed in secondary outcomes.

**Conclusions:**

Beta‐alanine supplementation is efficacious in augmenting muscle carnosine (+54% from mean baseline value) without side effects in patients with COPD in comparison with PL. However, accompanied beneficial changes in exercise capacity, quadriceps function, and muscle oxidative/carbonyl stress were not observed.

## Introduction

Patients with chronic obstructive pulmonary disease (COPD) often suffer from extra‐pulmonary features, including loss of lower‐limb muscle strength and endurance.^1^ These functional muscular deficits are mainly caused by structural and metabolic alterations, such as a muscle fibre type shift towards a higher proportion of fast‐twitch fibres, mitochondrial dysfunction, and elevated basal and exercise‐induced oxidative/carbonyl stress.^1^ These alterations decrease the muscle's oxidative capacity and cause earlier and greater reliance on the glycolytic metabolism, which in turn leads to earlier onset of muscle acidosis and contractile fatigue during exercise.^2^

Carnosine, an endogenous histidine‐containing dipeptide abundantly present in skeletal muscle,^3^ prominently in fast‐twitch fibres,^4^ might hold potential to delay onset of muscle acidosis and counteract oxidative/carbonyl stress in patients with COPD. Carnosine is formed by combining beta‐alanine (BA) with l‐histidine by carnosine synthase^3^ and is known to be lowered in patients with severe to very severe COPD.^5^ Carnosine plays different roles in the myocellular homeostasis: (i) carnosine is a natural antioxidant and interacts with and scavenges reactive oxygen species,^3^ thereby reducing production of reactive aldehydes due to lipid peroxidation.^6^ Furthermore, carnosine is able to quench these reactive aldehydes by forming conjugates,^7^ thus preventing formation of advanced glycoxidation and lipoxidation end‐products^8^; (ii) carnosine acts as pH buffer and is estimated to be responsible for 4–9% of intramuscular buffer capacity.^9^ Hence, carnosine is able to delay onset of muscle acidosis during high‐intensity exercise in healthy adults.^10^

Beta‐alanine, an affordable over‐the‐counter available nutritional supplement, has been proven to augment muscle carnosine and in turn exercise capacity in healthy older adults in a safe and feasible manner.^11^, ^12^, ^13^, ^14^, ^15^, ^16^, ^17^ Indeed, 12 weeks of BA supplementation (3.2 g/day) augmented muscle carnosine concentration by 85% from baseline value and in turn enhanced maximal (+12.2%) and submaximal (+36.5%) exercise capacity in healthy older adults between 60 and 80 years of age.^11^

To date, the efficacy of BA supplementation has not been studied in patients with COPD. Therefore, this study investigated BA supplementation in patients with COPD. It was hypothesized that 12 weeks of BA supplementation in patients with COPD would lead to muscle carnosine concentration augmentation, as well as accompanied beneficial effects on exercise capacity, quadriceps function, and muscle oxidative/carbonyl stress.

## Methods

### Study design and participants

This is a double‐blind, randomized, placebo (PL)‐controlled trial (clinicaltrials.gov identifier: NCT02770417) on the efficacy of 12 weeks of BA supplementation in patients with COPD. See supporting information for sample size calculation, randomization, and blinding. Participants were recruited between June 2016 and November 2018. The study was approved by the Ethics Committees of Jessa Hospital (Hasselt, Belgium) and Hasselt University (Diepenbeek, Belgium) (Belgian study registration number: B243201628086) and performed in accordance with the latest revision (2013) of the Declaration of Helsinki. Some baseline data comparing patients with COPD with age‐matched and sex‐matched healthy controls have been published before.^5^, ^18^

Patients with mild to very severe COPD, according to the Global Initiative for Chronic Obstructive Lung Disease (GOLD) guidelines,^19^ aged between 40 and 80 years were recruited at the Respiratory Medicine Department outpatient consultation of Jessa Hospital (Hasselt, Belgium). A clinicaltrials.gov registry deviation regarding the inclusion criteria of severity of airway obstruction is reported. Inclusion of patients with moderate to very severe airway obstruction was changed to mild to very severe airway obstruction, as muscle dysfunction is present throughout the whole disease spectrum.^20^, ^21^ See supporting information for exclusion criteria. All participants provided written informed consent prior to study inclusion.

### Intervention

Beta‐alanine and PL supplements were provided by Natural Alternatives International (NAI, USA). After a 2 week baseline assessment, the intervention group received 12 weeks of sustained‐release BA supplementation (SR CarnoSyn®) consisting of an oral BA intake of 3.2 g/day (four pills of 800 mg/day).^11^ The control group received maltodextrin as PL supplementation. See supporting information for details.

### Outcomes

Participants were assessed on 4 days in a 2 week period before the start of the intervention (baseline assessment) and during the last 2 weeks of the intervention (outcome assessment) (Supporting Information, *Table* *S1*).

Age, sex, smoking status, number of hospitalizations in the previous 12 months, COPD Assessment Test, modified Medical Research Council scale for dyspnoea, Charlson Comorbidity Index, and medication use were obtained. See supporting information for cut‐off scores, references, and description of assessment of pulmonary function, body composition, cycling capacity, walking capacity, physical activity (PA), quadriceps function, compliance, side effects and exploratory outcomes.

At baseline, a muscle biopsy of the middle part of m. vastus lateralis (right leg) was performed via the Bergström technique. For the outcome assessment, a biopsy was obtained by making a new incision at ±1 cm next to the baseline assessment incision. A part of the muscle sample was snap frozen in liquid nitrogen for muscle carnosine and related metabolites analysis via high‐performance liquid chromatography (HPLC) and for oxidative/carbonyl stress analysis via western immunoblotting. Another part was embedded in an optimum cutting temperature compound (FSC22 Frozen Section Media, Leica Biosystems, Richmond, IL, USA) and frozen in isopentane (VWR Chemicals, Radnor, PA, USA) cooled by liquid nitrogen for analysis of muscle fibre characteristics via immunostaining. To investigate systemic carnosine‐related metabolites (plasma histidine, BA, taurine, and serum carnosinase activity), two fasted venous blood samples (one serum and one lithium heparin plasma tube) were obtained. Muscle and blood samples were stored at −80°C in University Biobank Limburg until analysis. See supporting information for protocols.

Clinicialtrial.gov registry deviations regarding the outcomes are reported in the supporting information.

### Statistical analyses

The Statistical Package for the Social Sciences (v.24.0, IBM, NY, USA) was used for all statistics. Baseline assessment data are described as mean ± standard deviation or median (quartile 1–quartile 3), as appropriate after testing for normality using the Shapiro–Wilk test and for homogeneity of variance using Levene's test. Proportions are expressed in percentages. Comparison of compliance and side effects between the BA and PL groups was performed using the Mann–Whitney *U* test, and comparison of proportions between the BA and PL groups was performed using *χ* ^2^ test for homogeneity or Fisher's test, as appropriate. A linear 2 × 2 mixed model analysis was used to investigate the effect of BA supplementation on muscle carnosine and related metabolites, physical capacity, oxidative/carbonyl stress, and exploratory outcomes in comparison with PL. Data are expressed as mean [95% confidence interval]. *P* < 0.05 was set for significance. See supporting information and *Table* *S2* for details.

## Results

**Figure 1:**
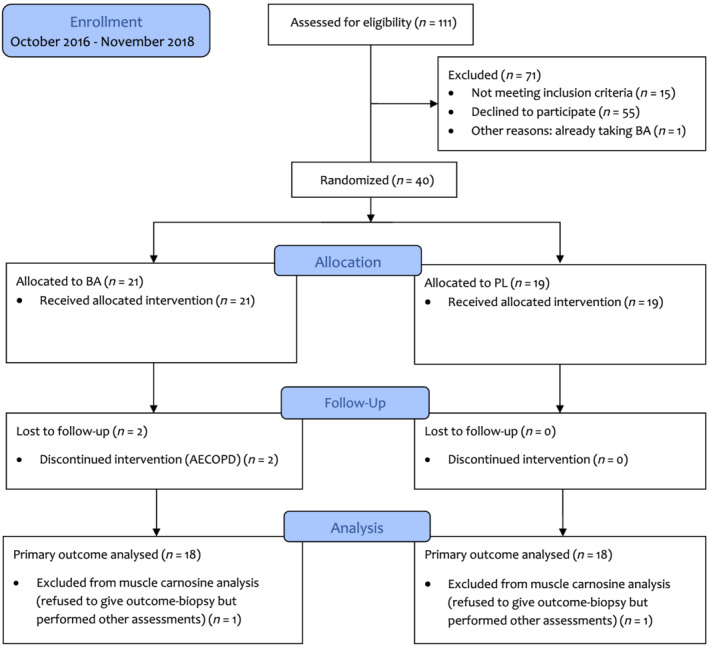
CONSORT flow diagram of the study. AECOPD, acute exacerbation of chronic obstructive pulmonary disease; BA, beta‐alanine; PL, placebo.

Baseline characteristics for both groups are shown in *Table* 1. Participant inclusion at each stage of the study is depicted by a CONSORT flow diagram (*Figure* 1). A 5% overall drop‐out rate was established (BA: 10 vs. PL: 0%).

**Table 1:** Baseline characteristics

	Beta‐alanine (*n* = 21)	Placebo (*n* = 19)
**Characteristics**		
Age (years)	66 ± 5	65 ± 6
Gender (*N*[%male])	16[76]	14[74]
Weight (kg)	71.9 ± 13.7	75.2 ± 12.4
BMI (kg/m^2^)	25.2 (21.2–29.6)	25.8 (22.9–28.8)
Whole‐body LMI (kg/m^2^)^a^	18.6 (15.1–20.2)	18.6 (17.0–19.8)
Whole‐body LMI under 10th percentile (*N*[%])^a^	1[5]	0[0]
Smoking status: S, EX, NS (*N*[%])	9[43], 11[52], 1[5]	7[37], 12[63], 0[0]
Hospitalization within previous 12 months: 0, 1, >1 (*N*[%])	16[76], 4[19], 1[5]	18[95], 1[5], 0[0]
COPD Assessment Test (pt)	15 ± 5	12 ± 7
COPD Assessment Test ≥ 18 points (*N*[%])	7[33]	4[21]
mMRC dyspnoea score (pt)	1 (1–2)	1 (0–2)
mMRC dyspnoea score ≥ 2 points (*N*[%])	5[24]	5[26]
Charlson Comorbidity Index (*N*)	2 (1–3)	2 (2–3)
Charlson Comorbidity Index ≥ 2 (*N*[%])	12[57]	15[79]
**Lung function**		
FEV_1_ (L)	1.56 ± 0.57	1.56 ± 0.40
FEV_1_ (%predicted)	55.2 ± 17.3	55.6 ± 10.0
FEV_1_/FVC (%)	46.1 (38.4–62.5)	50.4 (44.7–54.7)
TLC (%predicted)	117.3 ± 18.2	117.5 ± 13.5
RV (%predicted)	181.2 ± 47.8	175.5 ± 33.6
DLCO SB (%predicted)	54.2 (41.2–59.7)	51.6 (46.0–63.8)
GOLD Stage: I, II, III, IV (*N*[%])	3[14], 8[38], 8[38], 2[10]	0[0], 14[74], 5[26], 0[0]
**Medication use**		
Inhalation: short, long, long + ICS (*N*[%])^b^	0[0], 11[55], 9[45]	1[5], 11[58], 7[37]
Maintenance dose OCS or antibiotics (*N*[%])	4[19]	1[5]
Cholesterol (*N*[%])	9[43]	12[63]
Beta‐blocker (*N*[%])	5[24]	5[26]
Other cardiac (*N*[%])	7[33]	13[68]
Anti‐anxiety or anti‐depression (*N*[%])	3[14]	4[21]
Anti‐coagulants or anti‐aggregation (*N*[%])	8[38]	10[53]
Total number of medications (*N*[%])	5 (3–8)	6 (4–7)
**Muscle fibre characteristics** ^**c**^		
CSA ST fibre (μm^2^)	4386 (3558–5144)	4804 (3899–6281)
CSA FT fibre (μm^2^)	4549 ± 1616	4613 ± 2000
CSA all fibres (μm^2^)	4619 ± 1580	4911 ± 1583
% ST fibres (%)	34.7 ± 10.4	43.0 ± 14.5
Abnormally low ST fibres < 27% (*N*[%])	4[25]	4[21]
% ST fibre area (%)	36.3 ± 11.6	46.5 ± 18.2

BMI, body mass index; COPD, chronic obstructive pulmonary disease; CSA, cross‐sectional area; DLCO SB, Diffusion capacity of the Lung for Carbon Monoxide Single Breath; EX, ex‐smoker; FEV_1_, forced expired volume in 1 s; FT, fast‐twitch; FVC, forced vital capacity; GOLD, Global initiative for chronic Obstructive Lung Disease; ICS, inhaled corticosteroids; mMRC, modified Medical Research Council; NS, non‐smoker; OCS, oral corticosteroids; RV, residual volume; S, smoker; SD, standard deviation; ST, slow‐twitch; TLC, total lung capacity.

Data are expressed as mean ± SD or median (quartile 1–quartile 3) or number [percentage] as appropriate.

^a^ Altered sample size due to not performing DXA scan due to hip prosthesis (BA: *n* = 21; PL: *n* = 18).

^b^ Altered sample size due to incomplete medical record (BA: *n* = 20; PL: *n* = 19).

^c^ Altered sample size due to poor quality and/or staining of muscle cross‐sections (BA: *n* = 16; PL: *n* = 19).

### Compliance with and side effects of beta‐alanine supplementation

Pill compliance was high and similar (BA: 100 (98–100) vs. PL: 98 (96–100)%; *P* = 0.294) between groups. Reported complaints regarding supplement intake and common non‐respiratory complaints were not different between groups (*Table* *S3*). During the intervention period, eight participants experienced an exacerbation of COPD (two participants dropped out, and six participants completed the study (hospitalization: *n* = 1)).

### Effects of beta‐alanine supplementation

#### Muscle carnosine and related metabolites

**Figure 2:**
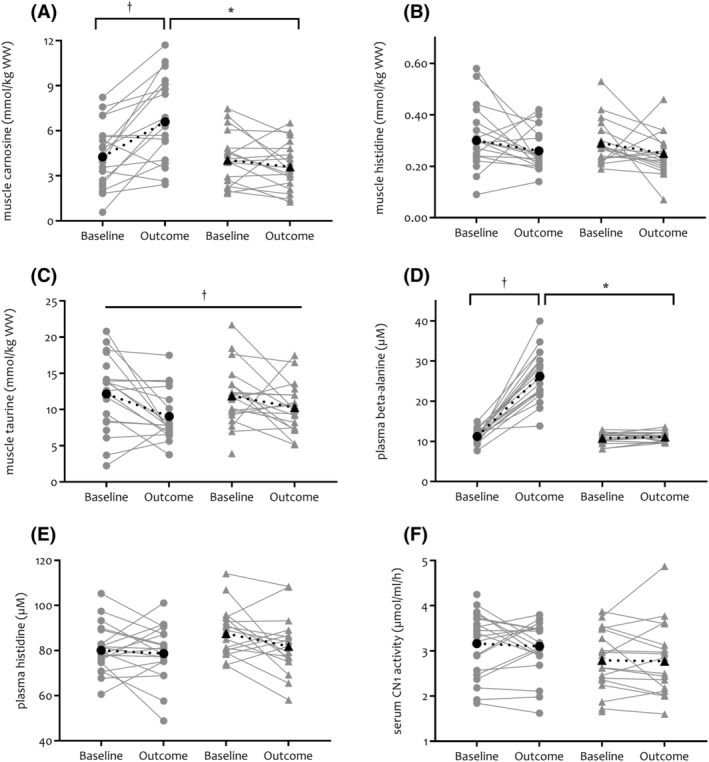
Effect of oral BA supplementation on muscle carnosine and related metabolites in muscle and plasma/serum in patients with chronic obstructive pulmonary disease. Muscle carnosine and related metabolites are depicted in Panels *(A)*–*(F)* (BA = circles, PL = triangles) by individual data points, and mean is shown in black and dotted line. Flat line = a main effect of time is present (†). Half tick‐down line = effects of time (†) and/or group (*) were explored separately by within (baseline vs. outcome within BA and PL) or between (BA vs. PL on baseline and outcome time point) analysis when a significant interaction group × time effect was present. BA, beta‐alanine; CN1, carnosinase; PL, placebo; WW, wet weight.

Twelve weeks of BA supplementation increased muscle carnosine concentration by +54% of the mean baseline value in comparison with PL (−12% of mean baseline value) in patients with COPD (BA baseline: 4.25 [3.32–5.17]; BA outcome: 6.59 [5.61–7.56] vs. PL baseline: 4.06 [3.09–5.04]; PL outcome: 3.59 [2.60–4.58] mmol/kg wet weight (WW); *P* < 0.001; *Figure* 2A). Within the BA group, the increase in muscle carnosine concentration was not different between patients with COPD in GOLD stage I/II (+2.39 ± 1.91 mmol/kg WW; +47% of mean baseline value; *n* = 10) and GOLD stage III/IV (+2.03 ± 2.83 mmol/kg WW; +54% of mean baseline value; *n* = 8; *P* = 0.757; *Figure* *S1*). No change was found for muscle histidine (*P* = 0.954) and taurine (*P* = 0.431) concentration after BA supplementation in comparison with PL, but a significant overall decrease in taurine concentration (time effect *P* = 0.016) was seen (*Figure* 2C).

Systemic carnosine‐related metabolites analysis showed that plasma BA concentration was increased after BA supplementation (+133% of mean baseline value) in comparison with PL (+3% of mean baseline value) (*P* < 0.001) (*Figure* 2D). No changes were found after BA supplementation in comparison with PL for plasma histidine concentration (*P* = 0.292) or serum carnosinase activity (*P* = 0.820) (*Figure* 2E and 2F).

Within the BA group, no correlations of baseline muscle carnosine and plasma BA with delta muscle carnosine were found (*Figure* *S2*). Additionally, no correlation of delta plasma BA with delta muscle carnosine was seen (*Figure* *S2*).

#### Physical capacity

Maximal and submaximal cycle capacity, walking capacity (6 min walking distance), quadriceps function, and PA showed no change after BA supplementation in comparison with PL. Even when participants with a baseline constant work rate cycle test (CWRT) time to exhaustion (TTE) of 20 min were excluded (BA: *n* = 6; PL: *n* = 9), no change after BA supplementation in comparison with PL was found. All details can be found in *Table* 2.

**Table 2:** Effect of oral beta‐alanine supplementation on physical capacity

	Beta‐alanine Baseline	Beta‐alanine Outcome	Placebo Baseline	Placebo Outcome	Interaction effect	Time effect	Group effect
**Cycling capacity**	***n* = 20**	***n* = 17**	***n* = 19**	***n* = 19**			
VO_2_peak‐CPET (mL/kg/min)^a^	17.6 [15.4–19.9]	17.4 [15.5–19.2]	18.2 [15.9–20.4]	17.4 [15.5–19.3]	0.384	0.088	0.851
Wpeak‐CPET (W)^a^	89 [75–103]	90 [77–102]	96 [81–110]	91 [79–104]	0.103	0.197	0.646
TTE‐CWRT (s)	820 [665–975]	703 [539–866]	910 [751–1069]	765 [606–924]	0.782	**0.014**	0.450
TTE‐CWRT (s) without participants who reached 20 min at baseline^b^	657 [512–801]	588 [434–742]	650 [479–820]	558 [387–728]	0.858	0.215	0.844
**Walking capacity**	***n* = 21**	***n* = 19**	***n* = 19**	***n* = 19**			
6MWD (m)	509 [473–545]	507 [471–543]	503 [465–541]	507 [469–545]	0.645	0.871	0.907
**Muscle function**	***n* = 21**	***n* = 19**	***n* = 18**	***n* = 18**			
Isometric quadriceps strength corrected for lean mass right leg (Nm/kg)	19.4 [17.9–20.8]	19.1 [17.6–20.5]	18.6 [17.1–20.2]	18.6 [17.0–20.1]	0.724	0.613	0.540
Isokinetic quadriceps endurance‐total work corrected for lean mass right leg (J/kg)^c^	151 [133–169]	148 [129–166]	154 [135–173]	152 [133–172]	0.839	0.500	0.772
**Physical activity**	***n* = 19**	***n* = 17**	***n* = 17**	***n* = 17**			
Step count (steps/day)	6464 [4623–8305]	6106 [4662–7549]	4974 [3028–6921]	4620 [3127–6113]	0.996	0.417	0.184
MVPA (min/day)	32 [20–44]	26 [13–38]	13 [0–26]	10 [−3 to 23]	0.689	0.164	**0.042**

6MWD, 6 min walking distance; CPET, cardiopulmonary exercise test; CWRT, constant work rate cycle test; J, joule; MVPA, moderate to vigorous physical activity; Nm, Newton metre; TTE, time to exhaustion; VO_2_, volume oxygen consumption; W, wattage.

Data are expressed as mean [95% confidence interval]. *P*‐values in bold are significant at *P* < 0.05.

^a^ Altered sample size due to absence of a participant on the CPET test moment (PL outcome: *n* = 18).

^b^ Altered sample size due to exclusion of participants who reached 20 min at baseline (BA baseline: *n* = 14; BA outcome: *n* = 12; PL baseline: *n* = 10; PL outcome: *n* = 10).

^c^ Altered sample size due to invalid isokinetic test due to incorrect execution (BA baseline: *n* = 16; BA outcome: *n* = 13; PL baseline: *n* = 14; PL outcome: *n* = 13).

#### Oxidative and carbonyl stress

**Figure 3:**
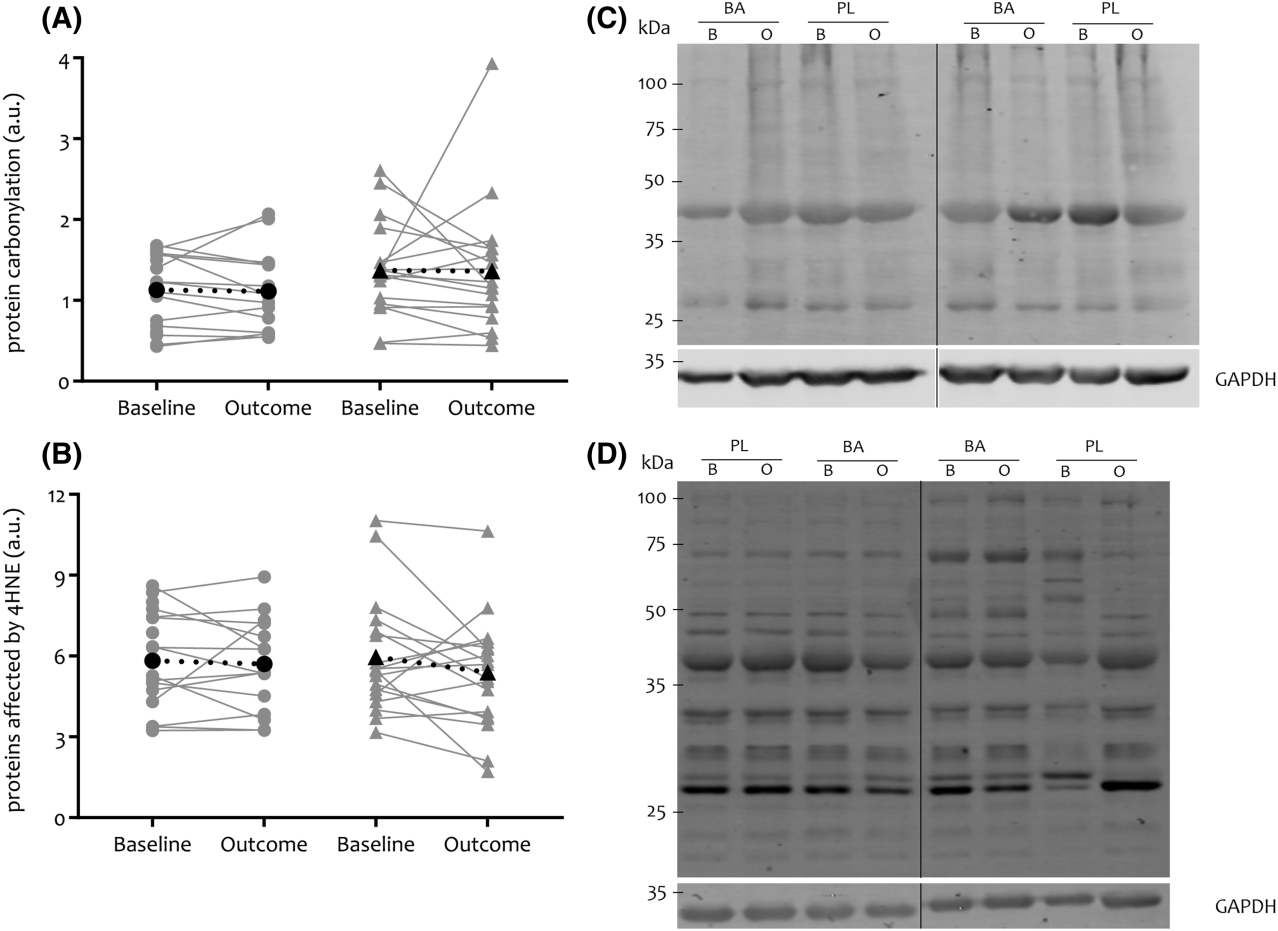
Effect of oral BA supplementation on oxidative and carbonyl stress in patients with COPD. Muscle proteins affected by carbonylation (Panels *A*–*C*) and 4HNE (*B*–*D*) (BA = circles, PL = triangles). Panels *(A)* and *(B)* show the quantification of muscle proteins affected by carbonylation and 4HNE relative to loading control GAPDH by individual data points, and mean is shown in black and dotted line. Panels *(C)* and *(D)* show a representative western blot (in total, 10 western blots were performed for both carbonylation and 4HNE) for muscle proteins affected by carbonylation and 4HNE, respectively. Baseline (B) and outcome (O) intervention samples of four patients with COPD (two patients in each group: BA/PL) were loaded per blot. Black vertical lines are lines where the blot was cut. 4HNE, 4‐hydroxynonenal; a.u., arbitrary units; BA, beta‐alanine; COPD, chronic obstructive pulmonary disease; GAPDH, glyceraldehyde 3‐phosphate dehydrogenase; kDa, kilodalton; PL, placebo.

Proteins affected by carbonylation (*P* = 0.966) and 4‐hydroxynonenal (4HNE; *P* = 0.320) (*Figure* 3) showed no change after BA supplementation in comparison with PL.

## Discussion

To the authors' knowledge, this is the first time BA supplementation has been investigated in patients with COPD. Twelve weeks of BA supplementation (3.2 g/day) did not cause side effects and was efficacious in augmenting muscle carnosine concentration (+54% of mean baseline value) in comparison with PL in patients with COPD. However, accompanied beneficial changes in exercise capacity, quadriceps function, and muscle oxidative/carbonyl stress were not observed.

Observed compliance with and absence of paresthesia after 12 weeks of BA supplementation in our study with patients with COPD is in line with the literature in young and older adults.^11^, ^12^, ^13^ Indeed, in two studies with older adults, no paresthesia was reported when using sustained‐release BA,^11^ the drop‐out rate was minimal,^13^ and compliance with supplement intake was high (>90%).^11^, ^13^

Twelve weeks of BA supplementation was efficacious in augmenting muscle carnosine concentration with +54% of mean baseline value in comparison with PL in patients with COPD. This positive response of muscle carnosine concentration is within the expected normal range (+40–60%) after BA supplementation in healthy adults across the age span.^22^, ^23^ This is also confirmed by Del Favero *et al*., the only other study that has quantified muscle carnosine concentration after BA supplementation in healthy older adults, who reported an +85% increase in muscle carnosine concentration using the same supplementation protocol as in our study.^11^ Interestingly, our research group showed that patients with severe to very severe COPD had a 33% lower muscle carnosine concentration in comparison with patients with mild to moderate COPD.^5^ It was observed that the muscle carnosine concentration increase after BA supplementation is similar in both severity groups, meaning patients with severe to very severe COPD manage to bring their muscle carnosine concentration back to and above normal after 12 weeks of BA supplementation (COPD: 5.80 vs. healthy controls: 4.64 mmol/kg WW).^5^ Additionally, our study shows that patients with mild to moderate COPD, who started the intervention with a normal muscle carnosine concentration,^5^ were also able to increase their carnosine concentration. It can therefore be hypothesized that baseline muscle carnosine concentration is not a strong determinant of response to BA supplementation in patients with COPD. This is supported by the lack of correlation in our study between baseline and delta muscle carnosine (*Figure* *S2A*) and by previous studies in healthy adults.^24^, ^25^

Individual muscle carnosine concentration response to BA supplementation showed variation (*Figure* 2A). This may be explained by the plasma BA response to BA supplementation as suggested by Blancquaert *et al*.^26^ An increase in plasma BA seems obvious in patients with COPD (*Figure* 2B), as BA uptake via the intestinal system and release in the circulation was found to be increased in all but one of the participants and increased on a group level to +133% of the mean baseline value. While this might seem evident, this is remarkable as fasted plasma samples were taken >8 h after the last supplement intake and pharmacokinetics (plasma BA is back to baseline after 5 h after acute BA supplement intake) and BA half‐life (after 80 min) are rather short.^27^ Our findings are further confirmed by those of Blancquaert *et al*., who found that after BA supplementation (6 g/day), fasted plasma BA was elevated the morning after last BA supplement intake on Day 12 (+123% of mean baseline value) and Day 23 (+169% of mean baseline value) of the supplementation period.^28^ Also, a study in vegetarians found that 800 mg of BA supplementation per day for 12 weeks resulted in an increase of 27.2% of fasted plasma BA.^29^ Additionally, they showed in mice (serum)^26^ and in humans (plasma) that change in fasted circulating BA determined change in muscle carnosine concentration after BA supplementation (soleus; *r* = 0.591; gastrocnemius; *r* = 0.442),^30^ suggesting that circulating BA is a good predictor of the amount of carnosine loading. These findings agree with the proposed plasma BA homeostasis hypothesis, which states that when plasma BA cannot be kept within homeostatic limits, BA is directed to transamination.^26^ Only when this pathway is saturated, BA will be transported into the myocyte and carnosine synthesis will occur. Thus, high responders to BA supplementation are likely individuals who fail to keep circulating BA between homeostatic limits. A correlation in similar magnitude between a change in plasma BA and a change in muscle carnosine after BA supplementation was however not observed in our patients with COPD (*r* = 0.373; *P* = 0.127). Whether this means that the proposed plasma BA homeostasis hypothesis does not apply in patients with COPD needs further corroboration, as m. vastus lateralis biopsies were taken in our study rather than from the soleus/gastrocnemius as in Blancquaert *et al*.,^28^ and our study was not powered to investigate this.

Muscle carnosine concentration augmentation after BA supplementation did not lead to an enhancement of our co‐primary (maximal and submaximal cycle capacity) or secondary (6 min walking distance, PA, and quadriceps function) physical capacity outcomes. However, based on the available literature in healthy older adults, enhancement of our co‐primary physical capacity outcomes was expected. Indeed, an increase in TTE on a maximal incremental exercise test (treadmill: +12.2%) and submaximal constant work rate exercise test (treadmill: +36.5% and cycle: +24.0%), and in physical working capacity at the fatigue threshold (cycling: +13.6–28.5%) has been reported.^11^, ^13^, ^14^, ^17^ In healthy (older) adults, an improved intramuscular pH‐buffer capacity is expected to be the reason why exercise capacity improves due to increased muscle carnosine concentration.^9^, ^10^ Patients with COPD suffer from earlier and greater reliance on the anaerobic metabolism during exercise, which in turn leads to muscle acidosis, elevated carbon dioxide levels, and an increased ventilatory load.^2^ Therefore, it was hypothesized that a muscle carnosine concentration augmentation would lead to an increased intramuscular buffering capacity to counteract the observed early muscle acidosis during exercise in patients with COPD. In turn, it was hypothesized that this would lead to a lower work of breathing and subsequently to a lower ventilatory load for the patient.^31^ We, however, did not find an improvement on the maximal and submaximal cycle test in patients with COPD after BA supplementation. A possible explanation for this is the high ventilatory load patients with COPD endure during exercise as a limiting factor.^32^ Based on our findings, whether a muscle carnosine concentration augmentation led to an increase in intramuscular buffering capacity remains speculative, as we did not measure the acidosis level during exercise. The capillary blood lactate response during maximal and submaximal cycle tests was assessed and was not different between the BA and PL groups (data not shown). Measurements of arterial blood gases would have been warranted to make assumptions on whether augmented carnosine was actually utilized as an intramuscular buffer.

It could also be speculated that carnosine was utilized within its role as an antioxidant during exercise. In our study, BA supplementation, however, did not show any beneficial effect on the expression of muscle proteins affected by carbonylation or 4HNE. The quenching role of augmented muscle carnosine after BA supplementation has already been proven in healthy adults by elevation of muscle carnosine‐conjugate formation with acrolein and 4HNE after acute high‐intensity exercise.^33^, ^34^ This was however not measured on a basal level and additional literature is scarce or even non‐existing in the older adult population. In fact, the current study is the first to have measured basal expression of muscle proteins affected by carbonylation or 4HNE, and our results should therefore be corroborated by future research.

Furthermore, not finding improvements in maximal and submaximal exercise capacity could also be due to methodological considerations. With regard to the BA supplementation intervention, we are convinced that the dosing strategy and intervention duration was sufficient to expect beneficial changes in exercise capacity among healthy subjects.^11^ The current data suggest this is not the case in patients with COPD. In relation to outcome methodology, the CWRT with a 20 min closed end is known to have a ceiling effect and could have impacted our findings. Nevertheless, sub‐analysis excluding patients with COPD who reached a CWRT TTE of 20 min at baseline also showed no improvements in TTE after BA supplementation (*Table* 2).

Investigating muscle function outcomes (quadriceps strength and endurance) also deserves attention, as patients experience a lower ventilatory burden during these exercises.^35^, ^36^ In our study, however, we did not observe any change in quadriceps isometric strength nor isokinetic endurance after BA supplementation. Studies in healthy older adults investigating muscle function have looked at functional tests and not at isolated quadriceps strength or endurance assessment.^11^, ^13^ Del Favero *et al*. showed no improvements in the Timed Up and Go test and the 30 s sit‐to‐stand test,^11^ while McCormack *et al*. showed a 22% improvement in the 30 s sit‐to‐stand test.^13^ Functional test results in healthy older adults are scarce and inconsistent. In young adults and athletes, quadriceps strength and endurance results are also contradictory.^15^ Although the literature is contradictory, it can be hypothesized that quadriceps isometric strength would not benefit from a muscle carnosine augmentation after BA supplementation due to the short duration of assessment (i.e. it relies on the phosphocreatine energy system) and it is greatly determined by the cross‐sectional area of the involved muscles. For quadriceps isokinetic endurance, however, a change might have been expected when considering the working mechanism of muscle carnosine, as this endurance assessment induces muscle acidosis and contractile fatigue. A possible reason for not finding a change might be the implemented isokinetic protocol. A 20‐repetition knee extension/flexion protocol was executed at 180°/s (duration = 20 s) and induces fatigue. This protocol duration may have been too short to observe the full intramuscular buffering capacity of carnosine. This is also supported by a meta‐analysis, which found that exercise tasks lasting <60 s did not improve after BA supplementation due to muscle acidosis not being the primary limiting factor.^37^

When collating our primary and secondary physical capacity findings, it remains difficult to conclude how carnosine was utilized in the muscle during exercise and why no improvement was observed in physical capacity after BA supplementation in patients with COPD. However, not finding an improvement in quadriceps endurance, which is along with central adaptations a requisite to improve whole‐body endurance capacity, indicates that carnosine may have more predominant roles (e.g. reactive aldehyde quenching) than intramuscular buffering during exercise in patients with COPD.^33^, ^34^

### Study limitations and considerations for future research

Potential biases encountered in this study include the recruitment of stable patients at the outpatient pulmonology consultation, as shown by the moderate symptom burden and well‐preserved physical capacity (no participant walked <350 m^38^) and lean mass (one participant <10th percentile^39^) of the participants. This may thus limit the generalizability of our findings to, for example, patients with COPD starting pulmonary rehabilitation.

To take future steps towards the potential clinical use of BA supplementation, it may be necessary to perform a study including only patients with reduced exercise capacity and whom are halted by leg fatigue during exercise to investigate whether augmented carnosine by BA supplementation leads to improved pH‐buffer capacity and as a result improved exercise capacity. Furthermore, the addition of an exercise training stimulus (as part of pulmonary rehabilitation) might be needed alongside BA supplementation when aiming to improve exercise capacity. Bex *et al*. suggested that trained muscles load carnosine better than those that are untrained, possibly due to increased blood flow to contracting muscles.^40^, ^41^ A recent study by Nemezio *et al*., however, showed that muscle carnosine loading was equal in the active (deltoid) and inactive (quadriceps) limbs of paraplegic athletes after BA supplementation.^42^ As literature is contradictory, scarce, and the ‘untrained’ model used in both studies is different, it remains interesting to investigate whether BA supplementation is a promising exercise training aid in patients with COPD.^43^ If so, patients might be able to train at higher intensities leading to greater improvement in exercise capacity and ultimately quality of life.

## Conclusions

Twelve weeks of BA supplementation is efficacious in augmenting muscle carnosine (+54% increase of mean baseline value) without side effects in patients with COPD in comparison with PL and brings muscle carnosine concentration back to and above normal in carnosine‐deficient patients. However, accompanied beneficial changes in exercise capacity, quadriceps function, and muscle oxidative/carbonyl stress were not observed.

## Conflict of interest

Jana De Brandt, Wim Derave, Frank Vandenabeele, Pascal Pomiès, Laura Blancquaert, Charly Keytsman, Marina S. Barusso‐Grüninger, Fabiano F. de Lima, Martijn A. Spruit, and Chris Burtin declare that they have no conflict of interest. Maurice Hayot has received research grants from Bastide Medical, which are not related to the current project; personal fees from AstraZeneca for participation to scientific lectures; financial support for congress participation from SOS Oxygène, Eole Santé, Boehringer Ingelheim, GlaxoSmithKline, and AstraZeneca; and hospitalities during local scientific meetings from ALK‐Abelló, Actelion Pharmaceuticals France, Vifor Fresenius Medical Care Renal Pharma, Sanofi Aventis France, Novartis Pharma, LVL Medical Sud, Chiesi, and SOS Oxygene Mediterranee.

## Funding

De Brandt J. is funded by the Flemish government. The Research of FWO (Research Foundation ‐ Flanders) Aspirant De Brandt J. is sponsored by FWO, Grant #11B4718N. Burtin C. is supported by Limburgs Kankerfonds. De Lima F.F. is supported by São Paulo Research Foundation (FAPESP), Grant #2019/10744‐3.

## Supporting information


Supporting Information S1
