# Supplementary material for: Efficacy of 12 weeks oral beta‐alanine supplementation in patients with chronic obstructive pulmonary disease: a double‐blind, randomized, placebo‐controlled trial
Source: J Cachexia Sarcopenia Muscle. 2022 Aug 17;13(5):2361–72. doi: 10.1002/jcsm.13048 (PMC9530565; doi:10.1002/jcsm.13048)
Supplement: Supplementary file 1 — Supporting Information S1 [file JCSM-13-2361-s001.docx]

# **Supplemental material**

**Title:** Efficacy of 12 weeks oral beta-alanine supplementation in patients with COPD: A double**-**blind, randomized, placebo-controlled trial

**Journal name:** Journal of Cachexia, Sarcopenia and Muscle

**Authors:**  De Brandt J.^1,2^ (PhD), Derave W.^3^ (PhD), Vandenabeele F.^1^ (PhD, MD), Pomiès P.^4^ (PhD), Blancquaert L.^3^ (PhD), Keytsman C.^1,2^ (PhD, PT), Barusso-Grüninger M.S.^1,5^ (PhD, PT), de Lima FF.^1,6^ (PhD, PT), Hayot M.^4^ (PhD, MD), Spruit M.A.^7,8^ (PhD, PT) & Burtin C.^1,2^ (PhD, PT)

**Affiliations:**

^1^Hasselt University, Faculty of Rehabilitation Sciences, REVAL - Rehabilitation Research Center, Diepenbeek, Belgium

^2^Hasselt University, BIOMED - Biomedical Research Institute, Diepenbeek, Belgium

^3^Ghent University, Department of Movement and Sports Sciences, Ghent, Belgium

^4^PhyMedExp, University of Montpellier – INSERM – CNRS – CHRU Montpellier, Montpellier, France

^5^São Carlos Federal University - UFSCar, LEFiR - Spirometry and Respiratory Laboratory, São Carlos, Brazil

^6^São Paulo State University (UNESP), Faculty of Science and Technology, Department of Physical Therapy, Postgraduate Program in Physical Therapy, Presidente Prudente, SP, Brazil

^7^CIRO+, Department of Research and Education, Horn, The Netherlands

^8^Maastricht University Medical Centre, Department of Respiratory Medicine, NUTRIM School of Nutrition and Translational Research in Metabolism, Maastricht, The Netherlands

**Corresponding Author:**

Chris Burtin - [chris.burtin@uhasselt.be](mailto:chris.burtin@uhasselt.be)

## **Methods**

### *Sample size calculation*

Sample size calculation (G*Power 3.1.9.2)[1] for independent T-test (two-tailed, α = 0.05, allocation ratio 1:1), showed that a minimum sample size of n = 16 was required for each group (beta-alanine (BA) or placebo (PL)) to reach a statistical power (1-β) of 0.80 based on the study of del Favero *et al.*[2], where primary outcome muscle carnosine concentration (ES: 1.21, n = 12) and co-primary outcomes time-to-exhaustion on incremental (ES: 1.03, n = 16) and constant work rate exercise test (ES: 1.71, n = 7) were compared between older adults receiving BA or PL. McCormack *et al.*[3] performed a similar study and reported a drop-out rate of ±25%. Therefore, a sample size of n = 20 for each group was established in this study.

### *Randomization and blinding*

Participants were randomly allocated to 12 weeks of oral BA or PL supplementation via an online randomization tool (<https://sealed.envelope.com>) using an alternating block size of 4 and 6, and were stratified for sex (BA and PL group include an equal amount of males/females) by a researcher (CK) not involved in the assessment of participants and analysis of data. Blank pill containers were used and labelled with participant study ID, and both study assessors (JDB, MBG) and researchers involved with data analysis (JDB, PP, LB, FFdL) as participants were blinded to the allocated intervention.

### *Exclusion criteria*

Exclusion criteria were known instable cardiac, neurological and/or musculoskeletal diseases that precluded safe participation in an exercise test, history of drugs/alcohol abuse, vegetarianism (long-term vegetarians (>8 years) have lower muscle carnosine[4]), inability to understand the Dutch language, an acute exacerbation of COPD leading to a change in medication or hospitalization in the last six weeks, and/or participation in a pulmonary rehabilitation program in the previous 12 months.

### *Intervention*

The BA and PL supplement were provided by Natural Alternatives International (NAI, USA) and both batches tested negative for contamination from prohibited substances by an independent drug surveillance laboratory (LGC Science Inc, Lexington, UK). Patients were instructed to co-ingest supplements with meals, as this can beneficially influence muscle carnosine loading[5]. In addition to the supplement, participants received a personal diary to administrate their daily pill intake, side-effects, general health complaints, doctor visits and change in medication. Participants were also called every two weeks to assess problems with pill intake, recovery of muscle biopsy, general health status and to troubleshoot when participants encountered problems. At the sixth week, the telephone call was replaced by a home-visit if desirable (not obligatory).

### *Outcomes*

Participants were assessed on four days over a period of two weeks at ReGo, Rehabilitation and Health Centre, of Jessa Hospital (Hasselt, Belgium) and at the Rehabilitation Research Center (REVAL) of Hasselt University (Diepenbeek, Belgium) before the start of the intervention (baseline assessment) and during the last two weeks of the intervention (outcome assessment) (Table S1).

Table S1: Assessment scheme per test day.

|  | **Assessment** | **Baseline** | **Outcome** |
| --- | --- | --- | --- |
| **Test day 1** | Age | x |  |
|  | Smoking status | x | x |
|  | Number of hospitalizations within the previous 12 months | x |  |
|  | CAT | x | x |
|  | mMRC dyspnea | x | x |
|  | CCI | x |  |
|  | MFI | x | x |
|  | EQ-5D-3L | x | x |
|  | PFT | x |  |
|  | Medication use | x | x |
|  | Body height | x | x |
|  | Body weight | x | x |
|  | DXA | x | x |
|  | 6MWT (2x) | x | x |
|  | Physical activity (start to wear accelerometer from next day) | x | x |
|  | Quadriceps strength | x | x |
|  | Quadriceps endurance | x | x |
|  | Hand grip strength | x | x |
|  | 4MGS | x | x |
| **Test day 2** | Spirometry | x | x |
|  | CPET | x | x |
| **Test day 3** | Spirometry | x | x |
|  | CWRT | x | x |
| **Test day 4** | Fasted venous blood sampling | x | x |
|  | Muscle biopsy | x | x |

Abbreviations: CAT = COPD Assessment Test; mMRC = modified Medical Research Council Scale for dyspnea; CCI = Charlson Comorbidity Index; MFI = Multidimensional Fatigue Inventory; EQ-5D-3L = EuroQol – 5 dimensions – 3 levels; PFT = Pulmonary Function Testing; DXA = Dual-energy X-ray Absorptiometry; 6MWT = Six-Minute Walking Test; 4MGS = Four-Meter Gait Speed; CPET = Cardiopulmonary Exercise Test; CWRT = Constant Work Rate cycle Test.

#### General and clinical characteristics

Cut-off scores and/or references used:

- COPD Assessment Test (CAT): patients scoring ≥18 points were classified as highly symptomatic[6]. Data from two participants are missing for the outcome assessment due to drop-out (BA: n = 2).
- modified Medical Research Council scale for dyspnea (mMRC): patients scoring ≥2 points were classified as highly dyspnoeic[7]. Data from two participants are missing for the outcome assessment due to drop-out (BA: n = 2).
- Charlson Comorbidity index (CCI)[8]
- Multidimensional Fatigue Inventory (MFI)[9]. Data from two participants are missing for the outcome assessment due to drop-out (BA: n = 2).
- EuroQol Five Dimensions Health Questionnaire (EQ-5D-3L)[10]. EQ-5D-3L visual analogue scale (VAS) data from three participants are missing for the outcome assessment due to drop-out (BA: n = 2) or not filling in the VAS score (BA: n = 1). EQ-5D-3L index data from two participants are missing for the outcome assessment due to drop-out (BA: n = 2).

#### Pulmonary function testing

Most recent pulmonary function testing (PFT) results (median (quartile 1 – quartile 3) of 50 (27 – 86) days between PFT and start of study) from patients with COPD were obtained from their medical record, including post-bronchodilator spirometry, lung volumes and diffusion capacity for carbon monoxide (Master Screen Body and PFT, Jaeger, Carefusion, San Diego, CA, USA). PFT was performed according to American Thoracic Society/European Respiratory Society (ATS/ERS) guidelines[11-14] and results served as baseline PFT data. The assessment of spirometry before the cycle tests on test day two and three (SpiroUSB, Carefusion, San Diego, CA, USA)[15] was performed according to ATS/ERS guidelines for spirometry [12].

#### Body composition

Body height and weight were measured, and body mass index (BMI, kg/m^2^) was calculated. Whole-body dual-energy x-ray absorptiometry (DXA; Lunar DPXL, General Electric Company GE, Boston, MA, USA) was performed to assess whole-body lean mass index (lean mass/height²) and the lean mass of the right leg. Whole-body lean mass reference values of Ofenheimer *et al.* were used to calculate the percentage of participants under the 10^th^ percentile[16]. Data are missing from one participant of the PL group for the baseline and outcome assessment because the DXA scan was not performed due to hip prosthesis.

#### Cycling and walking capacity

Cycle capacity was assessed on an electronic cycle ergometer (Cycle 407, ERGO-FIT, Pirmasens, Germany). Spirometry was performed before every cycle test (SpiroUSB, Carefusion, San Diego, CA, USA) to obtain forced expiratory volume in 1 s (FEV_1_) in order to calculate indirect maximum voluntary ventilation (MVV)[12]. Maximal cycle exercise capacity was assessed using a maximal cardiopulmonary exercise test (CPET)[17] and performed as follows: Participants were asked to wear a rubber face mask (Hans Rudolph Inc., Shawnee, KS, USA) and be seated on the ergometer. After 3 min of resting, patients started a 3 min warm-up phase at the lowest wattage (10 W) with a cadence between 50 – 70 rotations per minute. Subsequently, work rate was manually increased each minute by 5 or 10 W until symptom-limited peak work rate was reached. The reason for stopping the test was asked and followed by 5 min recovery at the lowest wattage and lower cadence. Oxygen uptake (VO_2_), carbon dioxide production (VCO_2_) and ventilation were measured breath-by-breath and averaged over 30 s during all phases except during recovery (OxyconPro^TM^, Jaeger^TM^, Carefusion, San Diego, CA, USA). Oxygen saturation was measured continuously using a wrist-worn pulse oximeter (Wristox 3150, Nonin Medical Inc., Plymouth, MN, USA). Continuous 12-lead ECG (KISS^TM^Multilead, GE Medical systems, Freiburg, Germany) was monitored throughout the test and blood pressure was manually measured every 2 min. Submaximal cycle capacity was assessed using the constant work rate cycle test (CWRT) at 75% of peak work rate achieved at the baseline assessment CPET with a closed end of maximal 20 minutes[18] and performed on the same cycle ergometer as CPET with similar test procedures. Time to exhaustion (TTE) was used as the reported measure. CPET and CWRT data from one participant (BA group) are missing from baseline and outcome assessments due to invalid CPET at baseline assessment and therefore outcome assessment was not performed. Furthermore, CPET data from four other participants are missing for the outcome assessment due to drop-out (BA: n = 2), knee pain (BA: n = 1) or absence of participant at the CPET test occasion (PL: n = 1). CWRT data from three participants are missing for the outcome assessment due to drop-out (BA: n = 2) or knee pain (BA: n = 1). Walking exercise capacity was assessed via a six-minute walking test (6MWT) in a 30 meter hallway[19]; the best of two tests was used for analysis. Four-meter gait speed (4MGS) test was performed in line with the protocol of Kon *et al.* [20]. Walking exercise capacity data from two participants of the BA group are missing for the outcome assessment due to drop out.

#### Muscle function

Isometric quadriceps strength (QS) and isokinetic quadriceps endurance (QE) were assessed with a computerized dynamometer (Biodex System 3, Shirley, NY, USA). For isometric QS, participants performed three maximal voluntary contractions (MVC) held for five seconds and the best MVC was used. For isokinetic QE, participants performed 20 full out extension-flexion cycles at 180°/s and total work (Joule) was used. Both QS and QE were corrected for lean mass of the right leg. QS and QE data from one participant of the PL group is missing at the baseline and outcome assessments due to unknown lean mass of the right leg as DXA scan was not performed due to hip prosthesis. QS data from two participants are missing for the outcome assessment due to drop-out (BA: n = 2). Data from nine participants (BA: n = 5; PL: n = 4) with an invalid isokinetic QE test due to incorrect execution are missing for the baseline assessment and therefore outcome assessment was not performed. Data from four other participants are missing for the outcome assessment due to drop-out (BA: n = 2) or invalid isokinetic QE test (BA: n = 1; PL: n = 1). Isometric handgrip strength was assessed via digital handheld dynamometry (Jamar® Plus+, Performance Health, Warrenwille, IL, USA) in accordance to Spruit *et al*.[21]. Data from three participants are missing for the outcome assessment due to drop out (BA: n = 2) or not performing the handgrip strength assessment (PL: n = 1).

#### Physical activity

Physical activity (PA) was assessed for at least five consecutive days using a tri-axial accelerometer (wGT3X-BT, Actigraph, Pensacola, FL, USA)[22]. Participants were asked not to wear the accelerometer during bathing or showering. ActiLife software (Actigraph, Pensacola, FL, USA) was used to analyse PA data (epoch length: 60s) with the algorithm of Choi[23]. PA was expressed as steps per day and minutes in moderate to vigorous PA per day. Cut-off values for moderate and vigorous physical activity were 1952 and 5725 counts/min, respectively, based on the algorithm of Freedson[24]. Data from four participants are missing for the baseline assessment due to invalid PA measurement (less than two valid days (a valid day is defined as >8 hours wearing time per day)[25] and/or no weekend days) (PL: n = 1) or due to not wearing the accelerometer (BA: n = 2; PL: n = 1) at the baseline assessment. Therefore, outcome assessment was not performed for these participants. Data from two other participants are missing for the outcome assessment due to drop-out (BA: n = 2).

#### Compliance and side-effects

Compliance was calculated based on the number of pills left in the participant’s pill container on the last assessment day during outcome assessment. The percentage of compliance was calculated: (total number of taken pills/total number of pills that should have been taken) x 100. Side-effects of intervention were quantified by listing and clustering complaints related to pill intake and non-respiratory health based on bi**-**weekly telephone calls, the optional home visit at week six and the participant’s diary. Exacerbation frequency and accompanied medication therapy were similarly obtained.

#### Carnosine and related metabolites

**Muscle sample -** For muscle carnosine (primary outcome), histidine, beta-alanine and taurine analysis, on average 15 mg was cut off the snap frozen muscle samples at -20°C and stored again at -80°C until analysis. Determination of metabolite concentration in muscle homogenate and plasma was performed by means of reversed-phase high-performance liquid chromatography (HPLC). Firstly, muscle samples were prepared for homogenization. Muscle samples were dissolved in a phosphate buffered saline (PBS) solution with 10 mM EDTA salt (30 µL per 1 mg wet weight muscle tissue) for homogenization with TissueRuptor II (Qiagen, Hilden, Germany) (3 x 20s lysing/20s rest on ice at 20 Hz/s). Muscle homogenates were stored at -20°C until analysis. Secondly, muscle metabolites were quantified by means of reversed-phase HPLC[26]. Muscle homogenates were deproteinized using 35% sulfosalicylic acid (Sigma-Aldrich, St-Louis, MO, USA) and centrifuged (5 min at 14000 g). Deproteinized supernatant (5 µL) was mixed with 75 µL AccQ Fluor Borate buffer and 20 µL reconstituted Fluor Reagent from the AccQTag chemistry kit (Waters, Milford, MA, USA). The same method was applied to combined standard solutions of carnosine (Flamma, Milan, Italy), histidine, beta-alanine and taurine (Sigma-Aldrich, St-Louis, MO, USA). The derivatized samples were applied to a Waters Alliance HPLC system (Milford, MA, USA) comprised of an Xbridge BEH C18 column (2.5µm, 4.6 x 150mm) and fluorescence detector (excitation/emission wavelength: 250/395 nm). The column was equilibrated with buffer A (10% eluent A (Waters, Milford, MA, USA) – 90% H_2_O), buffer B (100% acetonitrile; Sigma-Aldrich, St-Louis, MO, USA) and buffer C (100% H_2_O) at a flow rate of 1 ml/min at room temperature. Obtained HPLC chromatograms were processed via HPLC software (Waters, Milford, MA, USA) by performing integration of area under the curve from standards and samples. Based on the standard curve, muscle metabolite concentrations were determined taking into account wet weight (WW) of muscle sample (expressed in mmol/kg WW). Data from four participants are missing for the outcome assessment due to drop-out (BA: n = 2) or refusal of outcome-biopsy (BA: n = 1; PL: n = 1). Muscle BA could not be reliably calculated from the HPLC chromatograms and is therefore not reported.

**Fasted blood samples -** Plasma histidine, beta-alanine and taurine were quantified by means of reversed-phase HPLC[26] as aforementioned**,** except for homogenization step and derivatization step which was slightly different as deproteinized supernatant (10 µL) was mixed with 70 µL AccQ Fluor Borate buffer and 20µL reconstituted Fluor Reagent from the AccQTag chemistry kit. Serum carnosinase activity was quantified via fluorometric assay according to the method described by Teufel *et al.*[27]*.* Briefly, the reaction was initiated by addition of L-carnosine (Flamma, Milan, Italy) to a serum sample and stopped after 10 min of incubation at 37°C by adding 600 mM trichloroacetic acid (TCA; Sigma-Aldrich, St-Louis, MO, USA). For controls, TCA was added before L-carnosine. After centrifugation (15 min at 4500 rpm), supernatant was added to a mixture of OPA (incomplete o-phthaldehyde with 0.2% beta-mercaptoethanol (BME); Sigma-Aldrich, St-Louis, MO, USA) and 4 M sodium hydroxide (Sigma-Aldrich, St-Louis, MO, USA) and fluorescence was determined after 40 min (excitation: 360 nm and emission: 465 nm) by a Microplate reader (Tecan, Männedorf, Switserland). Data from one participant in the BA group was missing for the baseline assessment as fasted blood sampling was not possible and therefore also not sampled at the outcome assessment. Data from three other participants were missing for the outcome assessment due to drop-out (BA: n = 2) or no obtainment of blood sample due to non-fasted state of participant (PL: n = 1). Plasma taurine could not be reliably calculated from the HPLC chromatograms and is therefore not reported.

#### Muscle oxidative and carbonyl stress and fiber characteristics

**Muscle oxidative and carbonyl stress** – *Preparatory steps:*  On average, 30 mg of muscle samples were prepared for homogenization by dissolving in 500 µl lysis buffer RP1 (NucleoSpin^®^ RNA/Protein kit, Machery-Nagel, Düren, Germany) and 1% BME solution in ceramic bead tubes (Lysing Matrix D, MP Biomedical’s, Santa Ana, CA, USA). Samples were homogenized (20s at 6 m/s, 1 min on ice, 20s at 6 m/s) with bead beater grinder and lysis system (FastPrep-24^TM^, MP Biomedical’s, Santa Ana, CA, USA) and kept on ice until protein extraction. Samples were cleared by passing five times through a 20 G needle, followed by centrifugation (5 min at 14000 g), transfer of supernatant, addition of 300 µl ethanol (96 – 100%) and centrifugation (10 min at 14000 g). Next, supernatant was kept aside on ice for protein extraction according to the protocol of NucleoSpin^®^ RNA/Protein kit.

*Western immunoblotting:* Protein carbonylation was quantified with the OxyBlot Protein Oxidation Kit (Merck-Millipore, Burlington, MA, USA). Briefly, 10 µl of protein extract was derivatized to 2,4-dinitrophenylhydrazone (DNP-hydrazone) by reacting with 10 µl 2,4-dinitrophenylhydrazine solution for 15 min. Then 7.5 µl of neutralization solution was added to the sample mixture. Further, western immunoblotting was performed. The derivatized samples were separated by SDS-polyacrylamide gel electrophoresis (SDS-PAGE) and transferred to Immobilon-P PVDF (Merck-Millipore, Burlington, MA, USA) membranes. After an overnight rabbit anti-DNP primary antibody incubation (1:150), followed by an anti-rabbit secondary antibody (Eurobio Scientific, France) incubation (1:30000), scanning of membranes (Odyssey, LI-COR, Lincoln, NE, USA) was performed to detect total protein carbonylation. Lipid peroxidation (4-hydroxynonenal, 4HNE) was also quantified via western immunoblotting. Fifteen µl of protein extract were separated by SDS-PAGE and transferred to Immobilon-P PVDF membrane. Similar as the procedure described above, determination of total lipid peroxidation was performed with primary goat anti-HNE antibody (Abcam, Cambridg, UK) (1:400) and secondary anti-goat antibody (1:30000; Eurobio Scientific, France). Determination of loading control glyceraldehyde 3-phosphate dehydrogenase (GAPDH) was performed with primary mouse anti-GAPDH antibody (1:5000; Sigma-Aldrich, St-Louis, MO, USA) and secondary anti-mouse antibody (1:30000; Eurobio Scientific, France). Scanned membranes were quantified via ImageJ software (NIH, University of Wisconsin, Madison, WI, USA). For each polyacrylamide gel, a protein extract from an independent subject was loaded in the first lane as calibrator. This calibrator lane was deleted from Figure 3 for easier visualization of the results. Data from three participants (BA: n = 2; PL: n = 1) for the baseline assessment are missing due to an insufficient amount of muscle sample to perform the analysis and therefore also not included at the outcome assessment. Data from four other participants are missing for the outcome assessment due to drop-out (BA: n = 2), refusal of outcome-biopsy (BA: n = 1) or an insufficient amount of muscle sample to perform the analysis (PL: n = 1).

**Muscle fiber cross-sectional area (CSA) and type** – Muscle samples embedded in optimum cutting temperature compound were cut in 12 µm cross-sections with a cryostat (Leica CM1900 and CM3050 S, Leica Biosystems, Nussloch, Germany) at -20°C. Immunofluorescence staining was only performed at baseline assessment cross-sections. Shortly*,* cross-sections were placed in a humid chamber at room temperature to acclimatize. Primary antibody staining (mouse monoclonal anti-myosin heavy chain (skeletal, slow); Sigma-Aldrich, St. Louis, MO, USA) was performed by adding the dilution (1:2000 in PBS) to each cross-section. After 90 min incubation the primary antibody was removed by three washes with 100 µl PBS per cross-section. Subsequently, 50 µl of secondary antibody (Alexa Fluor 546; ThermoFisher Scientific, Waltham, MA, USA) and Hoescht33258 (Sigma-Aldrich, St-Louis, MO, USA), both at 1:1000 in PBS, were added to each cross-section. After 60 min incubation in the dark, the secondary antibody was removed by three washes with PBS and by one extra wash with H_2_O. A drop of histology mounting medium (Fluoroshield^TM^, Sigma-Aldrich, St. Louis, MO, USA) was placed on each cross-section, followed by a cover glass (VWR, Radnor, PA, USA). Cross-sections were preserved at 4°C until microscopy scheduled the next day. Fluorescence microscopy was performed with Axio Imager M1 microscope (Carl Zeiss AG, Oberkochen, Germany) at 10x enlargement and visualized with AxioCamMRm CCD Camera (Carl ZeissAG, Oberkochen, Germany) and Axiovison 4 software (Carl Zeiss AG, Oberkochen, Germany). Analysis of images was performed via circumscribing of fibers in ImageJ software (NIH, University of Wisconsin, Madison, WI, USA) by two independent raters (SV and MA) and one supervisor (JDB). On average 159 ± 98 (range: 59 – 465) fibers were measured per cross-section and the following parameters were derived: CSA of slow-twitch, fast-twitch and total fibers (i.e.**,** mean of all the fibers regardless of their type), percentage of slow-twitch fibers, and percentage of slow-twitch fiber area (i.e.**,** relative area of the muscle occupied by type of fiber) according to Mannion *et al.*[28]. A percentage of slow-twitch fibers <27% was considered as abnormally low in accordance to Gosker *et al.*[29]. Data from five participants of the BA group were missing as quality and/or staining of muscle cross-sections was poor.

#### Clinicaltrials.gov registry deviations

Some clinicaltrials.gov registered outcomes were not included in this study due to the following reasons: 1) Technical problems with equipment to assess maximal inspiratory and expiratory mouth pressures due to a malfunctioning valve; 2) Venous blood was obtained before and after CPET and CWRT to provide a mechanistic explanation in case of improved exercise capacity following BA supplementation. As there was no improvement in exercise capacity, analysing the venous blood samples seems redundant; 3) Resting ECG, blood pressure and ankle-brachial index were obtained to screen for ECG and vascular abnormalities before exercise testing and is considered a safety assessment and not an outcome; 4) Lung function testing and Charlson Comorbidity Index were only obtained during baseline assessment and not during outcome assessment; 5) The modified Baecke Questionnaire and BREQ-2 about PA and exercise (subjective measure) were eventually not assessed due to already assessing objectively measured PA; and 6) The Hospital Anxiety and Depression Scale (HADS) data was not included due to recently discovering in collaboration with MAPI trust that the used Dutch translation of the HADS was invalid. To be completely transparent, baseline and outcome assessment data from the following outcomes that were included at clinicaltrials.gov but are deemed explorative, i.e. 4-meter gait speed, hand grip strength, CAT, mMRC, MFI, and EQ-5D-3L, are reported in Table S4.

### *Statistical analysis*

A linear 2x2 mixed model analysis was chosen to investigate the effect of 12 weeks oral BA supplementation on muscle carnosine and related compounds, physical capacity, oxidative/carbonyl stress and exploratory outcomes in comparison to PL. Group (BA and PL) and time (baseline and outcome) were allocated as fixed factors and participant ID as random factor. Best fitted repeated covariance structure was chosen based on the Schwarz’s Bayesian Criterion. See Table S2 for a list of used repeated covariance structure per outcome measure. Maximum Restricted Maximum Likelihood was chosen as estimation method. If a significant interaction time x group effect was present, an independent sample comparison between BA and PL at the baseline and outcome assessment time points and a paired-sample comparison between baseline and outcome assessment within the BA and PL groups were performed separately. If there was a significant group and/or time effect, but no significant interaction time x group effect, only the main effects of group and/or time were interpreted and no separate comparisons per group or time-point were performed. After performing a sensitivity analysis (linear 2x2 mixed model) on daylight (as proxy of seasonality), daylight was not different across time for BA and PL group. Thus, daylight was not included as a covariate when analysing PA. An explorative sub-analysis was performed in the BA group via independent T-test comparing delta value (outcome minus baseline value) of carnosine in patients with COPD in GOLD stage I/II vs. GOLD stage III/IV. Within the BA group, correlations between baseline muscle carnosine, delta muscle carnosine, baseline plasma BA and delta plasma BA were performed using Pearson correlation.

Table S2: Repeated covariance structure per outcome measure

| **Outcome measures** | **Repeated covariance structure** |
| --- | --- |
| Muscle carnosine | Compound Symmetry |
| Muscle histidine | Unstructured |
| Muscle taurine | Scaled Identity |
| Plasma histidine | Compound Symmetry |
| Plasma beta-alanine | Diagonal |
| Serum carnosinase activity | Compound Symmetry |
| Protein carbonylation | Compound Symmetry |
| Proteins affected by 4HNE | Compound Symmetry |
| VO_2_peak – CPET | Unstructured |
| WRpeak – CPET | Unstructured |
| TTE – CWRT | Compound Symmetry |
| 6MWD | Compound Symmetry |
| Steps | Unstructured |
| MVPA | Compound Symmetry |
| Daylight* | Scaled Identity |
| QS | Compound Symmetry |
| QE | Compound Symmetry |
| **Exploratory outcomes** |  |
| 4MGS | Compound Symmetry |
| HGS right | Compound Symmetry |
| HGS left | Compound Symmetry |
| CAT | Compound Symmetry |
| mMRC dyspnea | Compound Symmetry |
| EQ-5D-3L VAS | Compound Symmetry |
| EQ-5D-3L index | Scaled identity |
| MFI general fatigue | Compound Symmetry |
| MFI physical fatigue | Compound Symmetry |
| MFI decreased activity | Compound Symmetry |
| MFI decreased motivation | Compound Symmetry |
| MFI mental fatigue | Unstructured |

Abbreviations: 4HNE = 4-Hydroxynonenal; VO_2_ = Volume of Oxygen consumption; WR = Work Rate; CPET = Cardiopulmonary Exercise Test; TTE = Time To Exhaustion; CWRT = Constant Work Rate cycle Test; 6MWD = six-minute walking distance; MVPA = Moderate to Vigorous Physical Activity; QS = Quadriceps Strength; QE = Quadriceps Endurance; 4MGS = Four-Meter Gait Speed; CAT = COPD Assessment Test; mMRC = modified Medical Research Council; EQ-5D-3L = EuroQoL - 5 dimensions - 3 levels; VAS = Visual Analogue Scale; MFI = Multidimensional Fatigue Inventory; * = only performed for sensitivity analysis.

## **Results**

Table S3: Compliance with and side-effects of oral beta-alanine supplementation in patients with COPD

|  | **BA**  **(N = 21)** | **PL**  **(N = 19)** | ***P*-value** |
| --- | --- | --- | --- |
| **Pill compliance** |  |  |  |
| Supplementation days (N) | 84 (83 – 85) | 84 (83 – 85) | 0.592 |
| Supplement intake calculated objectively (N) | 332 (323 – 337) | 329 (320 – 346) | 0.830 |
| Compliance (%) | 100 (98 – 100) | 98 (96 – 100) | 0.294 |
| **Complaints regarding pill intake** |  |  |  |
| Vomiting (N[%]) | 0[0] | 1[5] | 0.475 |
| Difficulty swallowing (N[%]) | 2[10] | 6[32] | 0.120 |
| Paraesthesia (N[%]) | 0[0] | 0[0] | - |
| **Exacerbation during supplementation period** |  |  |  |
| AECOPD (N[%]) | 5[24] | 3[16] | 0.698 |
| Treatment with OCS (N[%] AECOPD) | 5[100] | 3[100] | - |
| Treatment with OCS + antibiotics (N[%] AECOPD) | 2 [40] | 2 [67] | 1.000 |
| **Non-respiratory complaints during supplementation period** |  |  |  |
| Gastro-intestinal (N[%]) | 8[38] | 9[47] | 0.750 |
| Musculoskeletal (N[%]) | 7[33] | 7[37] | 1.000 |
| Psychological/cognitive (N[%]) | 2[10] | 5[26] | 0.226 |
| General fatigue (N[%]) | 2 [10] | 2 [11] | 1.000 |
| Dermal (N[%]) | 0[0] | 3[16] | 0.098 |
| Facial/oral/dental (N[%]) | 4[19] | 0[0] | 0.108 |
| Cardiac (N[%]) | 1[5] | 1[5] | 1.000 |
| Urological (N[%]) | 1[5] | 1[5] | 1.000 |

Abbreviation: COPD = Chronic Obstructive Pulmonary Disease; AECOPD = Acute Exacerbation of COPD; OCS = oral corticosteroids; BA = Beta-Alanine; PL = Placebo.

*Exploratory outcomes*

The effects of BA supplementation on exploratory outcomes are provided in Table S4.

Table S4: Effect of oral beta-alanine supplementation on exploratory outcomes

|  | **Beta-alanine**  **Baseline** | **Beta-alanine**  **Outcome** | **Placebo**  **Baseline** | **Placebo**  **Outcome** | **Interaction effect** | **Time effect** | **Group effect** |
| --- | --- | --- | --- | --- | --- | --- | --- |
| **Exploratory outcomes** | **n = 21** | **n = 19** | **n = 19** | **n = 19** |  |  |  |
| 4MGS (m/s) | 1.25 [1.15 – 1.36] | 1.32 [1.21 – 1.42] | 1.31 [1.20 – 1.42] | 1.21 [1.11 – 1.32] | ***P* = 0.015** | *P* = 0.595 | *P* = 0.732 |
| HGS right (kg)^a^ | 36.9 [32.4 – 41.4] | 35.7 [31.2 – 40.2] | 38.3 [33.6 – 43.0] | 37.6 [32.9 – 42.3] | *P* = 0.693 | *P* = 0.139 | *P* = 0.607 |
| HGS left (kg)^a^ | 35.8 [31.5 – 40.2] | 35.1 [30.7 – 39.5] | 36.0 [31.4 – 40.6] | 36.2 [31.6 – 40.8] | *P* = 0.430 | *P* = 0.671 | *P* = 0.839 |
| CAT (pt) | 15 [12 – 18] | 15 [12 – 17] | 12 [9 – 15] | 13 [10 – 16] | *P* = 0.459 | *P* = 0.974 | *P* = 0.256 |
| mMRC dyspnea score (pt) | 1 [1 – 2] | 1 [1 – 1] | 1 [1 – 1] | 1 [1 – 2] | *P* = 0.658 | *P* = 0.952 | *P* = 0.947 |
| EQ-5D-3L VAS (pt)^b^ | 72 [67 – 77] | 75 [70 – 81] | 78 [72 – 83] | 78 [73 – 84] | *P* = 0.476 | *P* = 0.253 | *P* = 0.212 |
| EQ-5D-3L index (pt) | 0.782 [0.731 – 0.833] | 0.771 [0.718 – 0.825] | 0.719 [0.666 – 0.772] | 0.757 [0.703 – 0.810] | *P* = 0.366 | *P* = 0.608 | *P* = 0.146 |
| MFI general fatigue (pt) | 10 [9 – 12] | 9 [8 – 11] | 9 [7 – 11] | 8 [7 – 10] | *P* = 0.724 | *P* = 0.168 | *P* = 0.312 |
| MFI physical fatigue (pt) | 11 [9 – 12] | 11 [9 – 12] | 10 [8 – 11] | 10 [8 – 11] | *P* = 0.953 | *P* = 0.856 | *P* = 0.409 |
| MFI decreased activity (pt) | 11 [9 – 12] | 10 [8 – 12] | 9 [8 – 11] | 10 [9 – 12] | ***P* = 0.049** | *P* = 0.821 | *P* = 0.658 |
| MFI decreased motivation (pt) | 10 [8 – 11] | 10 [8 – 11] | 10 [8 – 11] | 9 [7 – 11] | *P* = 0.578 | *P* = 0.605 | *P* = 0.870 |
| MFI mental fatigue (pt) | 8 [7 – 10] | 8 [6 – 10] | 7 [5 – 9] | 8 [6 – 10] | *P* = 0.126 | *P* = 0.518 | *P* = 0.470 |

*Data are expressed as mean [95% Confidence Interval]. P-values in bold are significant at P < 0.05. Abbreviations: 4MGS = Four-Meter Gait Speed; HGS = Hand Grip Strength; CAT = COPD Assessment Test; mMRC = modified Medical Research Council; EQ-5D-3L = EuroQoL - 5 dimensions - 3 levels; VAS = Visual Analogue Scale; MFI = Multidimensional Fatigue Inventory; ^a^altered sample size due to absence of participant on HGS test moment (PL outcome: n = 18); ^b^altered sample size due to not filling in VAS (BA outcome: n = 18).*

**
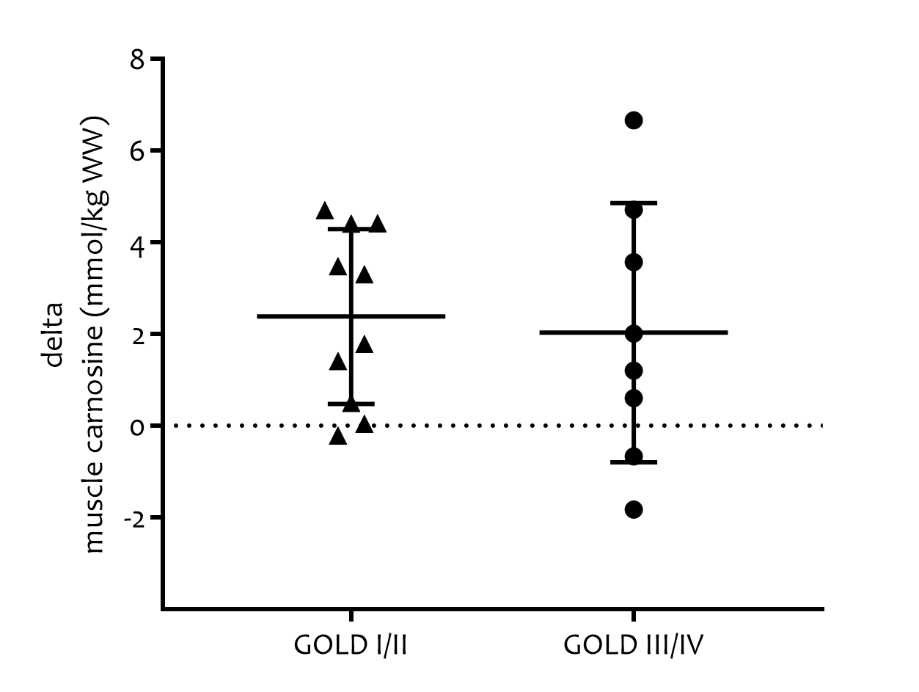
**

**Figure S1:** Delta muscle carnosine in patients with COPD in GOLD stage I/II vs. GOLD stage III/IV in BA group. Delta muscle carnosine is depicted as mean ± SD in patients with COPD in GOLD stage I/II vs. GOLD stage III/IV. Abbreviations: GOLD = Global initiative for chronic Obstructive Lung Disease; WW = Wet Weight.


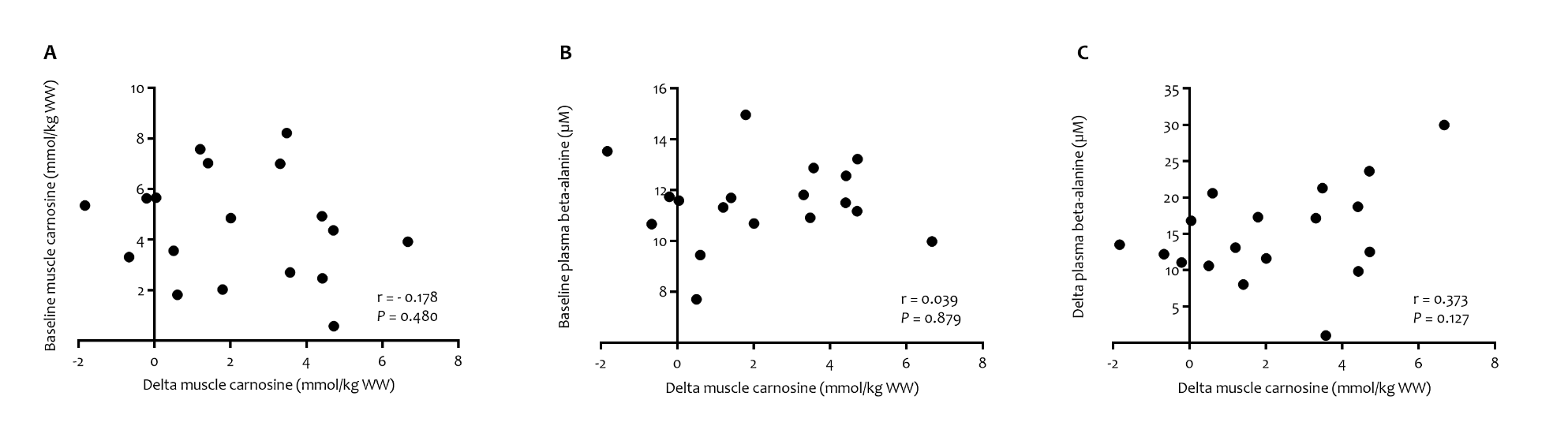


**Figure S2:** Correlations between baseline muscle carnosine and delta muscle carnosine (panel A), between baseline plasma beta-alanine and delta muscle carnosine (panel B), and between delta plasma beta-alanine and delta muscle carnosine (panel C). Abbreviations: WW = Wet Weight, µM = micro Molar.

# **References**

1. Faul F, Erdfelder E, Lang AG, Buchner A. G*Power 3: a flexible statistical power analysis program for the social, behavioral, and biomedical sciences. Behav Res Methods. 2007;39:175-91.

2. del Favero S, Roschel H, Solis MY, Hayashi AP, Artioli GG, Otaduy MC, et al. Beta-alanine (Carnosyn) supplementation in elderly subjects (60-80 years): effects on muscle carnosine content and physical capacity. Amino Acids. 2012;43:49-56.

3. McCormack WP, Stout JR, Emerson NS, Scanlon TC, Warren AM, Wells AJ, et al. Oral nutritional supplement fortified with beta-alanine improves physical working capacity in older adults: a randomized, placebo-controlled study. Exp Gerontol. 2013;48:933-9.

4. Everaert I, Mooyaart A, Baguet A, Zutinic A, Baelde H, Achten E, et al. Vegetarianism, female gender and increasing age, but not CNDP1 genotype, are associated with reduced muscle carnosine levels in humans. Amino Acids. 2011;40:1221-9.

5. Stegen S, Blancquaert L, Everaert I, Bex T, Taes Y, Calders P, et al. Meal and beta-alanine coingestion enhances muscle carnosine loading. Med Sci Sports Exerc. 2013;45:1478-85.

6. Smid DE, Franssen FME, Gonik M, Miravitlles M, Casanova C, Cosio BG, et al. Redefining Cut-Points for High Symptom Burden of the Global Initiative for Chronic Obstructive Lung Disease Classification in 18,577 Patients With Chronic Obstructive Pulmonary Disease. J Am Med Dir Assoc. 2017;18:1097 e11- e24.

7. GOLD. The Global Strategy for Diagnosis, Management and Prevention of COPD, 2022 report. 2022.

8. Charlson ME, Pompei P, Ales KL, MacKenzie CR. A new method of classifying prognostic comorbidity in longitudinal studies: development and validation. J Chronic Dis. 1987;40:373-83.

9. Smets EM, Garssen B, Bonke B, De Haes JC. The Multidimensional Fatigue Inventory (MFI) psychometric qualities of an instrument to assess fatigue. J Psychosom Res. 1995;39:315-25.

10. Rabin R, de Charro F. EQ-5D: a measure of health status from the EuroQol Group. Ann Med. 2001;33:337-43.

11. Macintyre N, Crapo RO, Viegi G, Johnson DC, van der Grinten CP, Brusasco V, et al. Standardisation of the single-breath determination of carbon monoxide uptake in the lung. Eur Respir J. 2005;26:720-35.

12. Miller MR, Hankinson J, Brusasco V, Burgos F, Casaburi R, Coates A, et al. Standardisation of spirometry. European Respiratory Journal. 2005;26:319-38.

13. Wanger J, Clausen JL, Coates A, Pedersen OF, Brusasco V, Burgos F, et al. Standardisation of the measurement of lung volumes. Eur Respir J. 2005;26:511-22.

14. Quanjer PH, Stanojevic S, Cole TJ, Baur X, Hall GL, Culver BH, et al. Multi-ethnic reference values for spirometry for the 3-95-yr age range: the global lung function 2012 equations. Eur Respir J. 2012;40:1324-43.

15. Berntsen S, Stolevik SB, Mowinckel P, Nystad W, Stensrud T. Lung Function Monitoring; A Randomized Agreement Study. Open Respir Med J. 2016;10:51-7.

16. Ofenheimer A, Breyer-Kohansal R, Hartl S, Burghuber OC, Krach F, Schrott A, et al. Reference values of body composition parameters and visceral adipose tissue (VAT) by DXA in adults aged 18-81 years-results from the LEAD cohort. Eur J Clin Nutr. 2020;

17. Radtke T, Crook S, Kaltsakas G, Louvaris Z, Berton D, Urquhart DS, et al. ERS statement on standardisation of cardiopulmonary exercise testing in chronic lung diseases. European Respiratory Review. 2019;28:180101.

18. van 't Hul A, Gosselink R, Kwakkel G. Constant-load cycle endurance performance: test-retest reliability and validity in patients with COPD. J Cardiopulm Rehabil. 2003;23:143-50.

19. Holland AE, Spruit MA, Troosters T, Puhan MA, Pepin V, Saey D, et al. An official European Respiratory Society/American Thoracic Society technical standard: field walking tests in chronic respiratory disease. European Respiratory Journal. 2014;44:1428-46.

20. Kon SS, Patel MS, Canavan JL, Clark AL, Jones SE, Nolan CM, et al. Reliability and validity of 4-metre gait speed in COPD. Eur Respir J. 2013;42:333-40.

21. Spruit MA, Sillen MJ, Groenen MT, Wouters EF, Franssen FM. New normative values for handgrip strength: results from the UK Biobank. J Am Med Dir Assoc. 2013;14:775 e5-11.

22. Van Remoortel H, Raste Y, Louvaris Z, Giavedoni S, Burtin C, Langer D, et al. Validity of six activity monitors in chronic obstructive pulmonary disease: a comparison with indirect calorimetry. PLoS One. 2012;7:e39198.

23. Choi L, Liu Z, Matthews CE, Buchowski MS. Validation of accelerometer wear and nonwear time classification algorithm. Med Sci Sports Exerc. 2011;43:357-64.

24. Freedson PS, Melanson E, Sirard J. Calibration of the Computer Science and Applications, Inc. accelerometer. Med Sci Sports Exerc. 1998;30:777-81.

25. Demeyer H, Burtin C, Van Remoortel H, Hornikx M, Langer D, Decramer M, et al. Standardizing the analysis of physical activity in patients with COPD following a pulmonary rehabilitation program. Chest. 2014;146:318-27.

26. Blancquaert L, Everaert I, Missinne M, Baguet A, Stegen S, Volkaert A, et al. Effects of Histidine and beta-alanine Supplementation on Human Muscle Carnosine Storage. Med Sci Sports Exerc. 2017;49:602-9.

27. Teufel M, Saudek V, Ledig JP, Bernhardt A, Boularand S, Carreau A, et al. Sequence identification and characterization of human carnosinase and a closely related non-specific dipeptidase. J Biol Chem. 2003;278:6521-31.

28. Mannion AF, Dumas GA, Cooper RG, Espinosa FJ, Faris MW, Stevenson JM. Muscle fibre size and type distribution in thoracic and lumbar regions of erector spinae in healthy subjects without low back pain: normal values and sex differences. J Anat. 1997;190 ( Pt 4):505-13.

29. Gosker HR, Zeegers MP, Wouters EF, Schols AM. Muscle fibre type shifting in the vastus lateralis of patients with COPD is associated with disease severity: a systematic review and meta-analysis. Thorax. 2007;62:944-9.
